# TRAINING PHYSICIAN-SCIENTISTS IN SOCIAL AND BEHAVIORAL SCIENCE: INDIANA ADRD MEDICAL SCIENTIST TRAINING PROGRAM

**DOI:** 10.1093/geroni/igac059.1968

**Published:** 2022-12-20

**Authors:** Nicole Fowler, Brittney-Shea Herbert, Christopher Callahan, Siyun Peng, Brea Perry, Karmen Yoder, Gary Landreth, William Truitt

**Affiliations:** Indiana University School of Medicine, Indianapolis, Indiana, United States; Indiana University School of Medicine, Indianapolis, Indiana, United States; Indiana University School of Medicine, IU Center for Aging Research, Indianapolis, Indiana, United States; Indiana University, Bloomington, Indiana, United States; Indiana University, Bloomington, Indiana, United States; Indiana University School of Medicine, Indianapolis, Indiana, United States; Indiana University School of Medicine, Indianapolis, Indiana, United States; Indiana University School of Medicine, Indianapolis, Indiana, United States

## Abstract

There is a critical need to grow and strengthen the pipeline of physician scientists who have expertise in sociomedical and behavioral research and are dedicated to addressing the nation's challenges posed by Alzheimer's disease and related dementias (ADRD). In 2021 The Indiana ADRD Medical Scientist Training Program (IADRD MSTP) was designed to meet this need and is built on the infrastructure of a robust portfolio of ADRD research, graduate training programs in medical neurosciences and sociology, and our existing MD-PhD program at Indiana University School of Medicine. The Aims of the IADRD MSTP are: 1) To recruit and train a competitive pool of diverse students who have an interest and commitment to social and behavioral research and patient care focused on ADRD; 2) To engage MD-PhD students early in mentored sociomedical and behavioral research that integrates IUs systems-based medical training curriculum with our cutting edge ADRD research that reinforces commitment and minimizes attrition of physician-scientists ADRD; and 3) To graduate students with dual MD-PhD degrees with strong methodological training in social and behavioral science and experts in ADRD who will be successful independent investigators at the best academic medical centers nationwide. The program includes rigorous didactic training in social, behavioral, and clinical research methods, with flexibility to allow students to focus their effort on one methodological area of interest; early initiation of ADRD research experiences with multidisciplinary teams of mentors and advisors; and the provision of educational experiences that enhance students' abilities to become independent researchers.

